# Isolation and Characterization of Two Squalene Epoxidase Genes from *Botryococcus braunii*, Race B

**DOI:** 10.1371/journal.pone.0122649

**Published:** 2015-04-01

**Authors:** Hidenobu Uchida, Koremitsu Sumimoto, Victor Marco Emmanuel Ferriols, Kenji Imou, Kiyotaka Saga, Kenichi Furuhashi, Shigeki Matsunaga, Shigeru Okada

**Affiliations:** 1 Laboratory of Aquatic Natural Products Chemistry, Graduate School of Agricultural and Life Sciences, The University of Tokyo, Yayoi, Bunkyo, Tokyo 113–8657, Japan; 2 Japan Science and Technology Agency-Core Research for Evolutional Science and Technology (CREST), Gobancho, Chiyoda, Tokyo 102–0076, Japan; 3 Institute of Aquaculture, University of the Philippines Visayas, Miagao, 5023 Iloilo, Philippines; 4 Laboratory of Biological and Mechanical Engineering, Graduate School of Agricultural and Life Sciences, The University of Tokyo, Yayoi, Bunkyo, Tokyo 113–8657, Japan; Mount Allison University, CANADA

## Abstract

The B race of the green microalga *Botryococcus braunii* produces triterpene hydrocarbons, botryococcenes and methylsqualenes that can be processed into jet fuels with high heating values. In this alga, squalene is also converted into membrane sterols after 2,3-epoxidation. In the present study, cDNA clones of two distinct squalene epoxidases (*BbSQE-I* and *-II*) were isolated. Predicted amino acid sequences encoded on these genes are 45% identical with each other. Introduction of *BbSQE-I* or -*II* into *Saccharomyces cerevisie erg1* mutants resulted in the complementation of ergosterol auxotrophy. The relative expression level of *SQE-II* increased 3.5-fold from the early stage to the middle phase of a culture period of 42 days, while that of *SQE-I* was almost constant throughout the culture period. Southern blot analyses suggested that these genes are single-copied genes. This is the first report on the isolation of functional *SQEs* that are encoded in duplicated loci in the algal genome.

## Introduction

The colonial green microalga *Botryococcus braunii* produces substantial amounts of hydrocarbons and is regarded as one of the most promising sources of algal biofuel. According to the types of hydrocarbons produced, *B*. *braunii* is classified into three races, namely A, B and L. Strains of race A produce *n*-alkadienes and *n*-alkatrienes [1], those of race B generate triterpenes in the form of botryococcenes and methylsqualenes [2], [3], and those of race L synthesize a tetraterpene as lycopadiene [4]. Among these different types of hydrocarbons, triterpenes from race B are thought to be the most promising source of biofuels because they can be processed into a fuel with higher octane numbers than those produced by race A [5]. Furthermore, hydrocarbon contents in race B, which can reach around 40%, and in some cases even more than 50% of algal dry weight, are much higher than those in race L.

**Fig 1:**
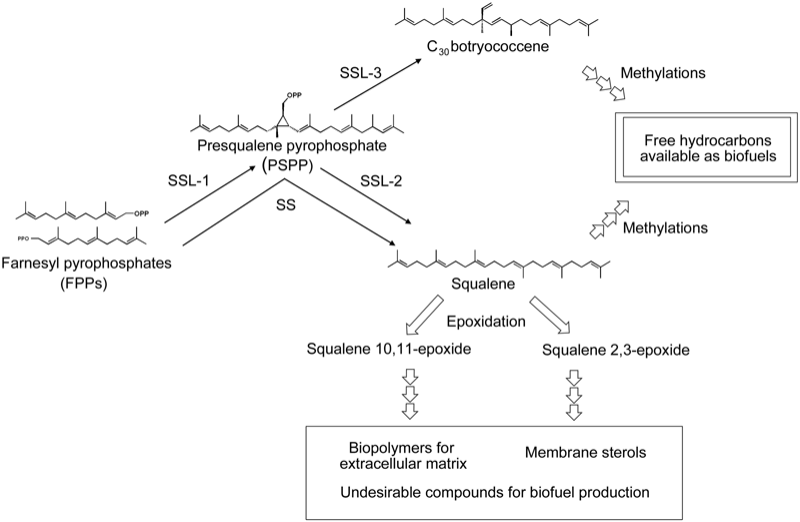
Biosynthesis and metabolism of triterpenes in *Botryococcus braunii*, race B. Squalene synthase (SS) converts two molecules of farnesyl pyrophosphate (FPP) into one molecule of squalene via presqualene pyrophosphate (PSPP). Squalene synthase-like protein (SSL) -1 (SSL-1) catalyzes formation of PSPP from two FPPs, SSL-2 converts PSPP into squalene, and SSL-3 synthesizes C_30_ botryococcene from PSPP. Squalene and C_30_ botryococcene are methylated and excreted outside cells as free hydrocarbons that can be used as biofuels. Squalene is epoxidized into squalene 2,3-epoxide that is the precursor of membrane sterols or into squalene 10,11-epoxide that is further converted into hydrophobic secondary metabolites including biopolymers.

In many eukaryotic organisms, one molecule of squalene, is produced from two molecules of farnesyl pyrophosphate (FPP). The enzymatic reaction of this process, which is performed by squalene synthase (SS), includes two steps of chemical conversions: formation of the intermediate, presqualene pyrophosphate (PSPP) and rearrangement of the carbon-carbon bond in a cyclopropane ring in PSPP to form C1’ to C1 condensation of two farnesyl residues [6]. The cDNA cloning of SS, which seems to be a conventional enzyme concerned with the production of squalene as the precursor for sterol biosynthesis, has been performed in *B*. *braunii* [7]. In addition to this SS enzyme, this alga was found to possess three distinct squalene synthase-like proteins, SSLs-1, 2, and 3 [8]. SSL-1 catalyzes only the first half of the reaction by SS, namely formation of PSPP from two molecules of FPP, while SSL-3 converts PSPP into C_30_ botryococcene with a C1’-C3 linkage of farnesyl residues (Fig 1). The C_30_ botryococcene is further methylated [9] into C_31_ to C_37_ botryococcenes [2] that are then excreted outside cells and deposited in the extracellular matrix as the major components of liquid oils. On the other hand, SSL-2 converts PSPP into squalene. There are therefore two distinct routes to synthesize squalene from FPP in the B race of *B*. *braunii*. Subsequently, some part of squalene is oxidized into squalene 2,3-epoxide and then metabolized into sterols as is in many eukaryotic organisms. As mentioned above, there are also C_31_-C_34_ methylsqualenes in the B race of the alga [3], [10] as secondary metabolites in addition to C_30_ squalene that can be used for sterol biosynthesis. In contrast to botryococcenes, these methylsqualenes exist merely as minor components in the liquid oil fraction. Some of the methylsqualenes are, however, epoxidized and further converted into various hydrophobic compounds such as unique carotenoids or even biopolymers that make up the extracellular matrix [11]. At the moment, it has not been uncovered if these methylsqualenes in the B race of *B*. *braunii* are derived from squalene molecules synthesized by the conventional SS or the combination of SSL-1 and SSL-2.

From the viewpoint of heating values of biofuels, introduction of oxygen atoms into molecules of hydrocarbons is definitely disadvantageous. Thus it is desirable to keep oils produced by the B race of *B*. *braunii* in the style of free extracellular hydrocarbons, such as botryococcenes or methylsqualenes by preventing introduction of oxygen atoms. In this context, the regulation of squalene epoxidases in *B*. *braunii* therefore presents itself as an important key point in order to attain commercial production of algal hydrocarbon oils.

In the present study, the authors have performed cDNA cloning of squalene epoxidase homologues from this alga, characterized their functions, and surveyed their gene expression patterns. Following considerations of evolutional implications for duplicated genes in triterpene-metabolism enzymes might unveil possible reasons why *B*. *braunii* evolved sophisticated triterpene biosyntheses pathways that are specific to this alga.

## Materials and Methods

### Culture

*Botryococcus braunii*, race B, Showa [12] was used in this study. Culture conditions were the same as previously reported [13].

### The cDNA cloning of *BbSQEs-I* and-*II*

Total RNA was isolated as previously reported [14]. The quality of total RNA was evaluated by A260/A280 ratio measured with a spectrophotometer and the integrity of ribosomal RNA bands was visualized by gel electrophoresis. In order to determine full-length sequences, overlapping *BbSQE-I* cDNA clones were isolated by initially amplifying an internal region by RT-PCR, followed by 5’ and 3’ rapid amplification of cDNA ends (RACEs) strategies. Initial sequence information for a *B*. *braunii* squalene epoxidase-like gene was obtained from an EST database [8] using the tBLASTn function with the protein sequence encoded on *Chlamydomonas reinhardtii* Cre15.g645100.t1.1 as a query sequence. This locus was detected in the phytozome 8 (http://phytozome.jgi.doe.gov/pz/portal.html#!info?alias=Org_Creinhardtii, accessed July 6, 2012). This resulted in a hit corresponding to the EST contig_14095. To amplify this contig by RT-PCR, the forward primer SQEI-internal-F and reverse primer SQEI-internal-R were subsequently designed. The nucleotide sequences of primers used in this study are listed in S1 Table. The template for RT-PCR was single strand cDNA which was synthesized using either oligo(dT) primer or random hexamers from the RNA of algae harvested at 6 days after inoculation into new medium. After obtaining a 0.3-kb internal fragment clone, 5’ and 3’ RACEs were performed using SMARTer RACE cDNA Ampification Kit (Clontech). For the 5’ RACE, the kit-supplied universal primer A mix was used as the forward primer SQEI-5RACE-F1 while the gene-specific SQEI-5RACE-R1 was used as the reverse primer. The 3’ RACE was performed using the gene-specific forward primer SQEI-3RACE-F and the kit-supplied universal primer A mix as the reverse primer SQEI-3RACE-R. For further extension of the cDNA 5’ end, another 5’ RACE was performed using a phagemid 5’ anchor primer as the forward primer SQEI-5RACE-F2 and the gene-specific reverse primer SQEI-5RACE-R2 with a previously described non-normalized cDNA library [7] as a template. For the construction of this library, total RNA was subjected to oligo(dT) column chromatography and the resulting poly(A) RNA was used as templates for single-stranded cDNA synthesis using oligo(dT) primer. From this, double-stranded cDNA was synthesized and packaged into lambda phage using ZAP-cDNA Synthesis Kit (Stratagene). RACE products were cloned and overlapping *SQE-I* sequences that cover the entire ORF were obtained. In the sequencing of every fragment, more than three independent plasmids were obtained, and all of these plasmids were subjected to double-strand sequencing to derive a consensus sequence. In order to obtain a full-length *SQE-I* cDNA clone, RT-PCR was further performed using the forward primer SQEI-full-F and the reverse primer SQEI-full-R. A 1.9-kb full-length ORF clone was finally obtained and is referred to as pSQEI.1903bp/pGEMTeasy.

Using RNA-seq data obtained from the algal samples harvested 17 days after inoculation into new culture medium, another squalene epoxidase homologue (*BbSQE-II*) was found by using known squalene epoxidase sequences as queries for a local BLAST search. This resulted in a hit corresponding to the RNA-seq contig_37075, which contained a tentative 1617-bp ORF. In order to isolate the corresponding full-length cDNA, the forward primer SQEII-full-F and the reverse primer SQEII-full-R were designed to include the tentative ORF and portions of the 5’ and 3’ UTRs. RT-PCR was performed using this set of primers and the single strand cDNA templates synthesized from 6-day old cultures as previously mentioned. Three independent clones were subjected to double-strand sequencing, and a consensus for *BbSQE-II* sequence was obtained. One of these clones is referred to as pSQEII.1848bp/pGEMTeasy.

### Complementation of yeast *erg1* mutant with *BbSQEs*

The ORF fragments of *BbSQEs-I* and-*II* were amplified by PCR using the full-length cDNA clones as templates. The primers SQEI-ORF-F and SQEI-ORF-R were used for *BbSQE-I* while the primers SQEII-ORF-F and SQEII-ORF-R were used to amplify *BbSQE-II*. The PCR product for *BbSQE-I* was digested with SpeI and SalI and subcloned into yeast expression vector pWV3 [15] to generate pSQEI.517aa/pWV3. The PCR product for *BbSQE-II* was digested with SpeI and XhoI and subcloned into pWV3 to generate pSQEII.538aa/pWV3. Double-strand nucleotide sequencing of pSQEI.517aa/pWV3 and pSQEII.538aa/pWV3 indicated that the amino acid sequences encoded on these clones had no amino acid substitutions as compared with corresponding protein sequences encoded on consensus *SQE* nucleotide sequences, although the 30th glutamate codon in pSQEI.517aa/pWV3 was synonymously substituted from gag to gaa, and the 195th alanine codon in pSQEII.538aa/pWV3 was synonymously changed from gac to gcg due to PCR errors. During subcloning, no artificial initiation codons were generated in front of the ORFs of *BbSQEs-I* and-*II*.

Resulting plasmids were introduced into the *Saccharomyces cerevisiae erg1* mutant KLN1 [16] using Frozen-EZ Yeast Transformation II Kit (Zymo Research). The pWV3 vector contains the *LEU2* selectable marker, and introduced cDNA is driven by a constitutive *ADH1* promoter. After introducing pSQEI.517aa/pWV3 or pSQEII.538aa/pWV3 into KLN1, transformant candidates were selected under anaerobic conditions on solidified—Leu SD medium supplemented with ergosterol. Colonies exhibiting *LEU2* phenotypes were selected. Introduction of *SEQ* plasmids into yeast cells were further checked by colony PCR using gene-specific ORF primers mentioned above. Vector controls were checked using the forward primer Vector-F and the reverse primer Vector-R. Selected yeast colonies were further streaked successively twice more onto—Leu SD medium containing ergosterol. In order to test for complementation of ergosterol auxotrophy, transformants were streaked on solidified YPD medium without ergosterol supplementation, and incubated at 30°C under aerobic condition for four days.

### Southern blot analyses

Genomic DNA was extracted from *B*. *braunii* cells using the cetyl trimethyl ammonium bromide (CTAB) method [17]. After digesting with restriction enzymes, Southern blot hybridization was performed according to a previous report [18]. For the detection of *BbSQE-I*, a DIG-labeled 325-bp probe was synthesized with the forward primer SQEI-probe-F and the reverse primer SQEI-probe-R using PCR DIG Probe Synthesis Kit (Roche). This probe fragment corresponds to the 325-bp region downstream from the 1278th base in the ORF. The *BbSQE-II* probe was similarly synthesized using the forward primer SQEII-probe-F and the reverse primer SQEII-probe-R. This probe fragment corresponds to the region ranging from 11th to 1487th base in the ORF. Hybridization was performed at 37°C using DIG Easy Hyb Granules (Roche). After stripping the *BbSQE-I* probe, the same membrane was re-hybridized with *BbSQE-II* probe. Hybridized genomic fragments were detected using a DIG Luminescent Detection Kit (Roche). Luminescent signal was detected using ImageQuant LAS4000 mini (GE Healthcare).

### RNA-seq analysis

Total RNA was isolated from algae harvested at 0 and 17 days after inoculation into new medium, and quality of isolated RNA was checked by A260/A280 ratio measured with a spectrophotometer and integrity of ribosomal RNA bands was visualized by gel electrophoresis. From 2 μg of total RNA isolated from day 0 culture, or the same amount of day 17 total RNA, cDNA libraries were constructed using Illumina RNA TruSeq Sample Preparation Kit. The 2.2 fmol of cDNA samples were loaded on an Illumina HiSeq to generate 101 bp paired-end reads (2 x 101). After trimming adaptor sequences, *de novo* assembly was performed using Trinity, released Feb. 25, 2013 [19]. Mapping was performed using Bowtie version 0.12.8 [20], and expression level was estimated using RSEM [21]. Tablet [22] was used to check mapped reads. Reads were deposited in DDBJ DRA with the accession numbers DRX026011 to DRX026014.

### Quantitative RT-PCR analysis

From aliquots of a liquid culture, total RNA was extracted in the same way described above and the quality was evaluated by A260/A280 ratio measured with a spectrophotometer, treated with Recombinant DNase I (Takara), subjected to phenol/chloroform extraction, precipitated with ethanol, and dissolved with distilled water. Using 5 ng of RNA as template, cDNA was synthesized using iScript Advanced cDNA Synthesis Kit for RT-qPCR (BioRad), and reacted with 10 μl of SsoFast EvaGreen Supermix (BioRad), according to manufacturer’s instructions. The reaction mixture of above mentioned cDNA synthesis kit includes both oligo(dT) and random primers. Primers to detect *BbSQEs-I* and-*II* were as follows: SQEI-qRTPCR-F, SQEI-qRTPCR-R; SQEII-qRTPCR-F and SQEII-qRTPCR-R. The expected PCR product sizes originating from either sets of SQE-I or SQE-II primers were both 103 bp. Annealing temperatures for above-mentioned primer sets were optimized using a thermal gradient block option. Efficiency of amplification was maximized after checking amplification curves (RFU vs. cylcles) and E values shown in standard curves (Cq vs. log starting quantity). Specificity of amplified PCR products for two primer sets, i.e. amplification of only 1 transcript in these reactions, was confirmed by observing a single peak in the melt peaks (-d(RFU)/dT vs. temperature). *B*. *braunii glyceraldehyde 3-phosphate dehydrogenase* gene (*BbGAPDH*) transcript was used as an internal standard for the calculation of relative amounts of *BbSQE* transcripts. As primers to amplify a 121-bp fragment of *BbGAPDH*, the forward primer GAPDH-qRTPCR-F and the reverse primer GAPDH-qRTPCR-R were used. The qRT-PCR was performed using CFX96 Real Time PCR Detection System coupled to a C1000 Thermal Cycler (BioRad). Cycling condition was as follows: 1 cycle of enzyme activation at 95°C for 30 sec, 45 cycles of denaturation at 95°C for 2 sec and annealing/extension at 56.0°C for 5 sec, and one cycle for melt curve determination, continuous from 65°C to 95°C in 0.5°C increments every 5 sec. Annealing temperature used for GAPDH-qRTPCR-F and -R was the same as that for SQEI-qRTPCRs or SQEII-qRTPCRs. This annealing temperature gave a best level of *GAPDH* amplification in RFU vs. cycle curve and a best E value in standard curve. Specificity of amplified PCR product for this primer set was also confirmed by observing a single peak in the melt peaks.

## Results and Discussion

### cDNA cloning and amino acid sequence similarity analyses

In order to isolate homologues of squalene epoxidase from *Botryococcus braunii*, Showa, available EST and RNA-seq data were initially surveyed from which two independent contigs were mined with a local BLAST function using known sequences of squalene epoxidase from other organisms as queries.

Through routine cDNA cloning procedures, two corresponding full-length clones, pSQEI.1903b/pGEMTeasy and pSQEII.1848bp/pGEMTeasy were isolated independently. Nucleotide sequencing of pSQEI.1903b/pGEMTeasy showed that this clone included a 1554-bp open reading frame (ORF), which was preceded by 318-bp 5’untranslated region (UTR) and followed by 31-bp 3’ UTR Clone pSQEII.1848bp/pGEMTeasy possessed a 1617-bp ORF, which was preceded by 57-bp 5’ UTR and followed by 174-bp 3’ UTR. The corresponding genes are referred to as *Botryococcus buranii squalene epoxidases-I* and-*II* (*BbSQEs-I* and-*II*). Nucleotide sequences of *BbSQEs-I* (AB923908) and-*II* (AB986538) were deposited in the DDBJ bank. Nucleotide sequences of the ORFs of *BbSQEs-I* and-*II* shared 56% homology while the predicted amino acid sequences of BbSQEs-I and -II shared 45% identity with each other.

**Fig 2:**
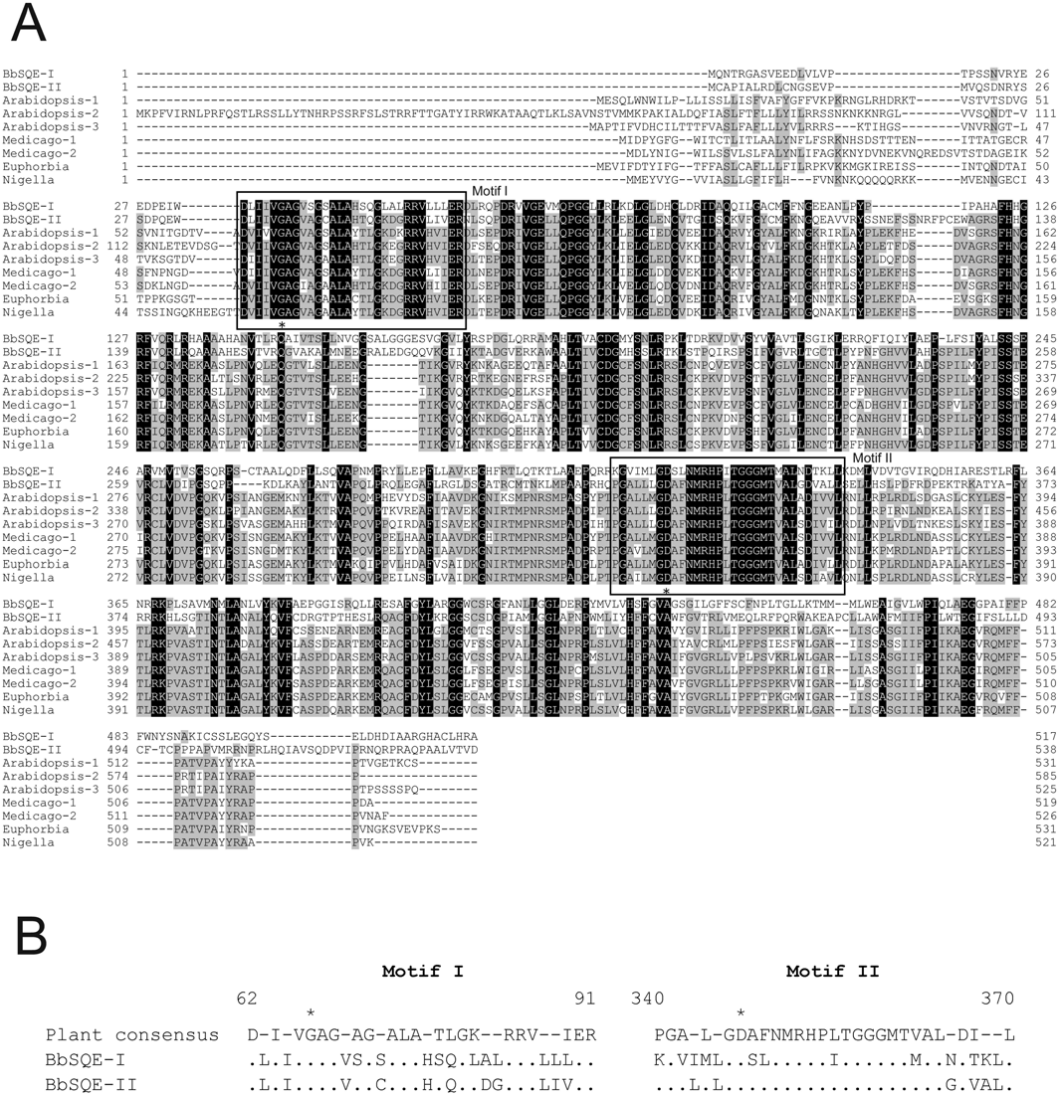
Amino acid sequences of BbSQEs-I and -II aligned with those from the other organisms. **A.** The amino acid sequences of land plant SQEs that complemented *Saccharomyces cerevisiae erg1* and BbSQEs were aligned using CLUSTAL W (ver.1.83) multiple sequence alignment tool (www.ebi.ac.uk/Tools/msa/clustalw2/help/faq.html, accessed Nov. 14, 2014) and adjusted manually. Amino acid residues that are 100% identical in the alignment are highlighted in black and those which are more than 50% identical are highlighted in grey. Boxes show conserved domains, hyphens denote the gaps in aligned sequences and asterisks indicate amino acid residues whose point mutations could result in the loss of complementation of *erg1* [26]. **B.** Sequences of motifs I and II in BbSQEs-I and -II aligned with plant consensus sequence. The top sequence shows the consensus among seven plant sequences from panel A. Numbers denote amino acid residue positions in AtSQE1. Hyphens indicate varieties in plant sequences. Asterisks are the same as in panel A. BbSQE sequences that are identical to the plant consensus are shown with dots.

In order to estimate the function of *BbSQEs-I* and *-II*, predicted amino acid sequences were compared to squalene epoxidases from land plants (Fig 2A), *Arabidopsis thaliana* (*AtSQE1-6*) [23], *Medicago truncatula* (*MtSEs1* and *2*) [15], *Nigella sativa* (*NsSQE*) [24] and *Euphorbia tirucalli* (*EtSE*) [25], which functionally complemented the *Saccharomyces cerevisiae erg1* mutant. The deduced amino acid sequence of BbSQE-I shared considerable identities with NsSQE1 (39%), EtSE (39%), AtSQE1 (37%), and MtSE1 (37%). The predicted amino acid sequence of BbSQE-II shared relatively higher identities with NsSQE1 (53%), AtSQE1 (52%), MtSE1 (52%), and EtSE (51%). The authors further compared BbSQE sequences of the two conserved domains [26], which are indispensible for NAD-binding (motif I) or substrate binding (motif II) in *S*. *cerevisiae* SQE. Complementation of *erg1* can be lost when a point mutation at the residue of either G_67_S, or D_347_F,W,P is introduced to the amino acid sequence of yeast ERG1 [26], where the numbering of amino acid residues are referred according to that in the AtSQE1 sequence shown in Fig 2B. At the 67th and 347th residues, BbSQEs-I and -II retained the conserved amino acids, G and D, respectively. As compared with consensus sequences among seven plant SQEs that complemented the *erg1* mutant, BbSQE-I had five amino acid substitutions at residues 71, 78, 79, 81 and 89, whereas BbSQE-II sequence possessed only two at residues 79 and 89. In motif II, BbSQE-I had eight amino acid substitutions at residues 340, 342, 344, 348, 349, 355, 362 and 367 as compared with the plant consensus sequence, while BbSQE-II sequence had only one substitution at residue 367. These results point to the higher degree of conservation of BbSQE-II in relation to those of functional plant SQE sequences compared to BbSQE-I.

### Functional characterization by yeast mutant complementation

**Fig 3:**
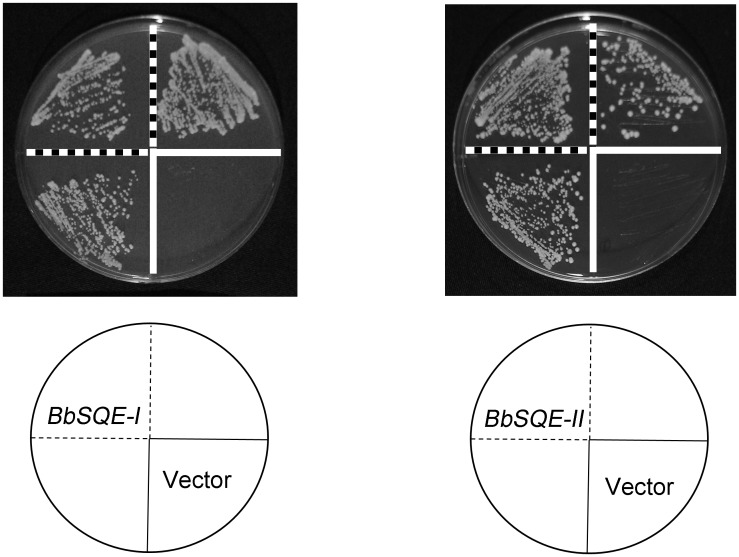
Complementation of KLN1 by *BbSQEs-I* and *-II*. The *BbSQE* cDNA clones were cloned into expression vector pWV3, and introduced into *Saccharomyces cerevisae erg1* mutant, KLN1. Under anaerobic conditions, transformants were selected on leucine-deficent synthetic minimum medium plates which were supplemented with ergosterol and re-streaked twice more on fresh plates. Subsequently, three lines of SQE transformants were streaked on solidified YPD medium and incubated for four days under aerobic conditions, along with an empty vector control.

In order to investigate the functions of *BbSQEs-I*, and-*II*, corresponding cDNA clones were introduced into the *Saccharomyces cerevisiae erg1* mutant KLN1 (Fig 3). KLN1 cells cannot grow on medium which is not supplemented with ergosterol due to disruption of the endogenous gene for squalene epoxidase [16]. When *BbSQE-I* was introduced into KLN1, the ergosterol auxotrophy in this mutant was restored. Under the same culture conditions, no growth was observed in the empty vector control. When analyzing *BbSQE-II* transformants, the same result was obtained. These results show that *erg1* was complemented by either *BbSQE-I* or *-II*, and that both *BbSQEs-I* and *-II* served as functional squalene epoxidase gene in the yeast cells. Based on these results, the authors present the first report of the occurrence of multiple functional squalene epoxidase genes from algae. Since *B*. *brunii* gene for NADPH: cytochrome P-450 reductase, which is indispensible for the function of squalene 2,3-epoxidase, is yet to be cloned and functionally characterized, *in vitro* enzyme characterization, including kinetic studies and substrate specificity of squalene epoxidases shall be performed in the future studies.

### Investigation and comparison of gene copy number

**Fig 4:**
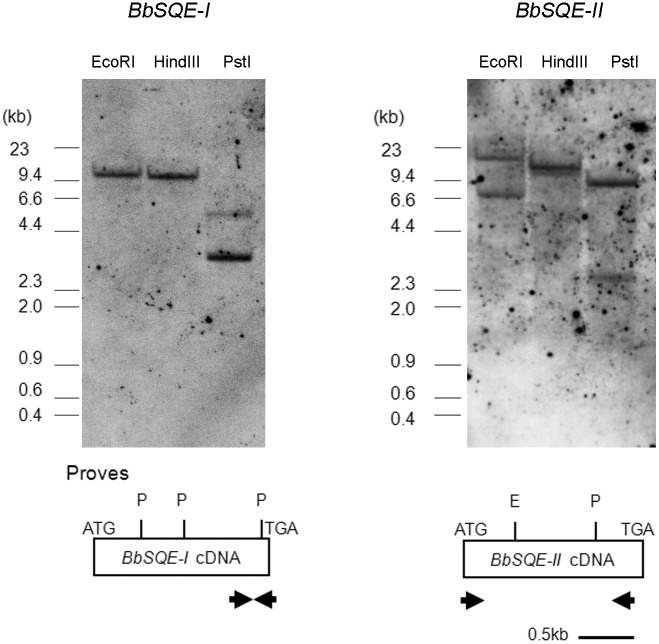
Southern blot analyses of *BbSQEs-I* and *-II* using genomic DNA. The cDNA fragments of 325-bp *BbSQE-I*, or 1477-bp *BbSQE-II* were labeled with digoxigenin, and used as probes for detecting corresponding genomic fragments. Upper panels show the Southern blots. After stripping probe of *BbSQE-I*, the same membrane was re-hybridized with that of *BbSQE-II*. The representative drawings at the bottom show positions of probe regions (arrows), as well as PstI and EcoRI sites (vertical bars with a letter) in the ORF (rectangles). Scale bar for cDNA length is indicated at the bottom.

In order to determine the gene copy number of *BbSQEs*, Southern blot analyses were performed (Fig 4). Using a 325-bp *BbSQE-I* cDNA fragment as a probe, two fragments with sizes of 5.5 kb and 3.3 kb were detected in genomic DNA digested with PstI. Considering that the exon region, which hybridizes with this probe, is divided into two fragments after PstI digestion, Southern blot detection of the same number of PstI-digested genomic fragments (exons plus introns) using the same probe suggests that *BbSQE-I* is a single copy gene. Detection of a 12-kb EcoRI or an 11-kb HindIII fragment using the same probe was consistent with this interpretation. When a 1477-bp *BbSQE-II* cDNA probe was used, two PstI genomic fragments of 2.8-kb and 8.7-kb were detected. As with the *BbSQE-I* probe, the *BbSQE-II* cDNA probe fragment includes a single PstI and a single EcoRI site. Thus, the Southern blot analyses on genomic DNA digested with either of these two enzymes showed two fragments. Therefore *BbSQE-II* also appeared to be a single-copy gene.

In the present study, the authors have cloned two *SQE* genes, and exhibited their functional complementation in the *Saccharomyces cerevisiae* ERG1-null mutant (Fig 3). Interestingly, this is the first report of the detection of plural SQEs in a green algal genome. According to BLAST searches on the NCBI database [27, 28] (http://blast.st-va.ncbi.nlm.nih.gov/Blast.cgi: accessed September 18, 2014), only a single SQE gene occurs in other green algae such as *Bathycoccus prasionos* RCC1105, *Micromonas pusilla* CCMP1545, *Micromonas pusilla* RCC299, *Ostreococcus lucimarinus* CCE9901, *Ostreococcus tauri* OTTH0595, *Coccomyxa subellipsoidea* C-169, and *Chlorella variabilis* NC64A. Similarly, a BLAST analyses on phytozome 10 (http://phytozome.jgi.doe.gov/pz/portal.html#!search?show=BLAST&method=Org_Creinhardtii: accessed September 18, 2014) revealed only a single locus assigned as a squalene epoxidase gene in either of the genomes of *Chlamydomonas reinhardtii* CC-505 Mt+ or *Volvox carteri f*. *nagariensis* Eve. As in many algal species, squalene epoxidase only occurs as a single copy gene in fungi [29], [30] and mammals [31], [32], [33]. The observed gene duplication of *BbSQE* in this study falls in line with previous reports of the occurrence of gene duplication in other genes involved in triterpene biosynthesis in *B*. *braunii* such as the gene for 1-deoxy-D-xylulose 5-phophate synthase (*DXSs-I*, *-II* and-*III*) [13] and squalene synthase-homologues, *SS* [7] and *SSLs-1*, *-2*, *-3* [8]. Gene duplication of key enzymes in triterpene biosyntheses will be suited to supply larger amounts of precursors for liquid triterpene hydrocarbon production in the alga. Considering the possible merits of the duplication of *BbSQE*, we can point out a unique metabolic pathway in *B*. *braunii*, race B. In this organism, a certain amount of squalene is methylated and secreted into its extracellular matrix as a component of hydrocarbon oils [3], [10], while a part of the squalene pool is metabolized into cell membrane sterols via squalene 2,3-epoxide as in the other eukaryotic organisms [34]. Throughout its evolutionary history, *B*. *braunii* might have acquired a new metabolic pathway to produce hydrocarbon oil through the duplication of genes, while retaining traditional pathways involved in cell division. Interestingly, the genome size for the B race of *B*. *braunii* is 166 Mb [35] and is mostly larger than those of other unicellular green algae examined so far, including Prasinophyceae, Chlorophyceae, and some of Trebouxiophyceae [36]. This observation is consistent with the duplication of genes which are involved in unique triterpene production in *B*. *braunii*.

### Gene expression analysis during a culture period

In order to compare absolute expression levels between *BbSQEs-I* and *-II*, RNA-seq analysis was performed using the RNA from the cells collected immediately after inoculation into new medium (day 0) and 17 days after inoculation (day17) (Table 1). Expression levels of *BbSQEs-I* and-*II* transcripts (expressed as fragments per kilobase of exon per million mapped sequence reads—FPKM) at day 0 did not differ significantly. Observations for samples collected at day 17 show a slight down-regulation of *BbSQE-I* while *BbSQE-II* exhibited considerable up-regulation from day 0. Furthermore, expression levels of *BbSQE-II* at day17 were about two times higher than that of *BbSQE-I*. These results show that the transcript amount of *SQE-II* is more abundant than that of *BbSQE-I* in the middle of the algal culture cycle.

**Table 1:** RNA-seq analysis of *BbSQEs-I and -II* at days 0 and 17 after inoculation into fresh culture medium.

	FPKM^a^	
Gene	Day 0^b^	Day 17^c^
*BbSQE-I*	7.30	5.33
*BbSQE-II*	7.59	10.21

^a^Fragments per kilobase of exon per million mapped sequence reads.

^b^Total number of aligned reads was 89,185,438.

^c^Total number of aligned reads was 57,605,684.

**Fig 5:**
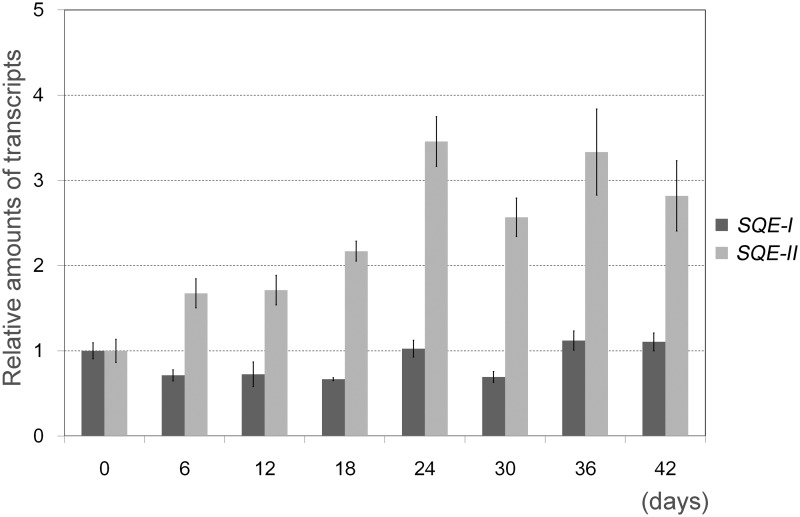
Expression of *BbSQEs-I* and *-II* during a culture peroid. *Botryococcus* culture was inoculated into fresh liquid medium and cultured for 42 days. Aliquots were harvested every 6 days and total RNA was extracted from each sample. Using qRT-PCR, relative amounts of *BbSQE-I* and-*II* transcripts were determined using that of *GAPDH* as a reference gene. The relative amounts at day 0 are shown as 1.0. Values are the mean of three technical replicates ± S.D. in samples collected every 6 days of a representative algal culture.

For more detailed analysis of *BbSQE* expression levels during a culture period, the authors further performed qRT-PCR analysis using RNA samples that were extracted from cells harvested at 6 day intervals after inoculation into new medium (Fig 5). Using primers specific for squalene epoxidases (*BbSQEs-I* and *II*) or glyceraldehyde 3-phosphate dehydrogenase (*BbGAPDH*), corresponding genes were amplified from cDNAs synthesized from RNA samples. Using *GAPDH* as a reference gene, relative transcript levels of *BbSQE-I* were almost constant throughout the culture period, with no changes greater than 35% of the initial levels at day 0. Relative amounts of *BbSQE-II* transcript were observed to increase considerably up until day 24 and the elevated transcript levels were maintained until the end of the culture period at day 42. The maximum relative transcript level of *BbSQE-II* was about 3.5-fold higher compared to that at day 0.

The qRT-PCR analysis of *SQEs* showed that relative transcript levels *BbSQE-I* were fairly constant while that of *SQE-II* increased as culture period proceeded. This expression pattern is distinct from those of the three genes of 1-deoxy-D-xylulose 5-phosphate synthase in *B*. *braunii* (*BbDXSs*) [13]. Relative transcript accumulation amounts of *BbDXSs-I*, *-II* and-*III* reach their maximum at an early stage of culture (day 6), and their levels go down from days 20 to 30. These observations suggest that enhanced expression of *BbSQE-II* occurs at latter stages of culture compared to the peaks of *BbDXSs-I*, *-II* and-*III*. A previous report indicated that botryococcene synthesis activity is highest at around day 6 [37] when the expression levels of *BbDXSs* reach their maximum. The results from this study suggest that the *BbSQE* gene expression in *B*. *braunii* might not be coupled with expression of *DXSs* given that DXSs, key enzymes in the MEP pathway [38], are involved in processes farther upstream from SQEs.

## Conclusions

cDNA clones coding for two distinct squalene epoxidases, *BbSQEs*-*I* and -*II*, were isolated from the B race of *Botryococcus braunii*. The predicted amino acid sequence of BbSQE-II showed higher similarity to those of plant SQEs than that of BbSQE-I. Introduction of these genes into the *Saccharomyces cerevisiae erg1* deficient mutant restored its ergosterol auxotrophy. Gene expression of *BbSQE-I* was almost constant during a 42 day culture period, while that of *BbSQE-II* increased during the latter half of the culture period. From these results, the two BbSQEs therefore seem to have different physiological functions for either algal growth or triterpene hydrocarbon metabolism.

## Supporting Information

S1 Table Primers used in this study. (XLSX)
